# American Medical Association

**Published:** 1885-05-20

**Authors:** 


### AMERICAN MEDICAL ASSOCIATION.

Our senior editor attended the late meeting of the above national medical body, and did himself the pleasure of mingling socially with the representative medical men of the country. There were about six hundred delegates present, and though the attractions of the great Exposition were calculated to divert the attention of delegates in some degree, yet there was a fair attendance upon the sittings and many interesting papers were presented.

The opening prayer was made by the distinguished Presbyterian divine, Dr. Palmer, and the address of welcome by Dr. Logan, of the City of New Orleans, in a most impressive and cordial manner.

Dr. Campbell's address was excellent and was well received.

In our next we will, perhaps, speak more fully of our trip and what we saw in the Crescent City.

### OFFICERS OF THE AMERICAN MEDICAL ASSOCIATION FOR NEXT YEAR.

President—William Brodie, M.D., of Michigan.
1st Vice-President—Samuel Logan, M.D., of Louisiana.
2d Vice-President—A. Y. P. Garnet, M.D., of District of Columbia.
3d Vice President—Charles Alexander, M.D., of Wisconsin.
4th Vice-President—W. F. Peck, M.D., of Iowa.
Permanent Secretary—W. B. Atkinson, M.D., of Pennsylvania.
Treasurer—R. J. Dunglison, Pennsylvania.
Librarian—C. H. A. Kleinschmidt, M.D., of District of Columbia.

